# Gut microbiota and hypertensive disorders in pregnancy: evidence from the Mendelian randomization study

**DOI:** 10.18632/aging.205019

**Published:** 2023-09-11

**Authors:** Xinrui Wu, Qi Li, Dihui Lin, Jiawang Cai, Houxiang Huang, Hongzhuan Tan

**Affiliations:** 1 School of Medicine, Jishou University, Jishou, China; 2 Xiangya School of Public Health, Central South University, Changsha, China; 3 Xiangxi Center for Disease Control and Prevention, Jishou, China

**Keywords:** hypertensive disorders in pregnancy, gut microbiota, pre-eclampsia, Mendelian randomization, causal relationship

## Abstract

Background: Recent studies have shown that gut microbiota (GM) is related to hypertensive disorders in pregnancy (HDP). However, the causal relationship needs to be treated with caution due to confounding factors and reverse causation.

Methods: We obtained genetic variants from genome-wide association studies including GM (N = 18,340) in MiBioGen Consortium as well as HDP (7,686 cases/115,893 controls) and specific subtypes in FinnGen Consortium. Then, Inverse variance weighted, maximum likelihood, weighted median, MR-Egger, and MR.RAPS methods were applied to examine the causal association. Reverse Mendelian randomization (RMR) and multivariable MR were performed to confirm the causal direction and adjust the potential confounders, respectively. Furthermore, sensitivity analyses including Cochran’s Q statistics, MR-Egger intercept, MR-PRESSO global test, and the leave-one-out analysis were conducted to detect the potential heterogeneity and horizontal pleiotropy.

Results: The present study found causalities between eight gut microbial genera and HDP. The HDP-associated gut microbial genera identified by MR analyses varied in different subtypes. Specifically, our study found causal associations of *LachnospiraceaeUCG010, Olsenella, RuminococcaceaeUCG009*, *Ruminococcus2*, *Anaerotruncus*, *Bifidobacterium*, and *Intestinibacter* with GH, of *Eubacterium* (*ruminantium group*), *Eubacterium* (*ventriosum group*), *Methanobrevibacter*, *RuminococcaceaeUCG002*, and *Tyzzerella3* with PE, and of *Dorea* and *RuminococcaceaeUCG010* with eclampsia, respectively.

Conclusions: This study first applied the MR approach to detect the causal relationships between GM and specific HDP subtypes. Our findings may promote the prevention and treatment of HDP targeted on GM and provide valuable insights to understand the mechanism of HDP in different subtypes from the perspective of GM.

## INTRODUCTION

Hypertensive disorders in pregnancy (HDP), defined as elevated blood pressure that occurs for the first time during pregnancy, are serious complications that affect 4.1-19.4% of pregnant women globally [1]. After years of prevention and intervention, HDP remains the second leading cause of maternal and prenatal mortality [2]. Although various mechanisms including oxidative stress [3], chronic uterine placental ischemia [4], immune dysregulation [5], and vascular endothelial dysfunction [6] have been studied, the explicit pathogenesis of HDP has not been fully elucidated.

Gut microbiota (GM) has been observed to change significantly during gestation and is crucial for maintaining host physiology and homeostasis [7]. Mounting evidence demonstrated the gut microbiota dysbiosis in HDP patients. For example, Chen et al. reported that PE (pre-eclampsia) patients have a lower diversity of GM with some beneficial genera reduced such as *Faecalibacterium* and *Akkermansia* [8]. Another nested case-control study demonstrated the difference in GM composition in early pregnancy between HDP patients and healthy controls [9]. However, these findings had some limitations. Firstly, the environment of the human intestine is very complex and often affected by various factors, some covariates that cannot be measured may cause confounders. Secondly, most existing results came from observational studies, the timing of exposure and outcome remains unclear and it’s easy to cause reverse causal association. Furthermore, the previous studies are mainly focused on PE patients, ignoring other subtypes which have different pathogenesis and degree of organ damage. Therefore, it is crucial to explore the possible causal association between GM and HDP in specific subtypes after confounders and reverse causation well controlled.

Mendelian randomization (MR) is a useful method for causal inference using genetic variants (e.g. Single Nucleotide Polymorphisms, SNP) as instrumental variables (IVs) [10]. Because the alleles from parents to offspring are randomly assigned, freely combined and the genotypes remain stable after birth. MR is regarded as the “most natural” randomized controlled trial (RCT), and its advantages such as reducing confounding factors as well as excluding reverse causality provide an effective way for causal inference based on observational studies [11, 12]. Furthermore, the ability and accuracy of genetic variants detection in genome-wide association studies (GWAS) have been greatly improved, and the measurement error has been reduced compared to conventional research [13]. Many studies have used MR analysis to explore the correlation between GM and some complex human diseases [14–16]. Therefore, our study performed a bidirectional multivariable MR analysis using the GWAS summary statistics to detect the causal relationship between GM and different subtypes of HDP, which may provide novel insights to understand the mechanism of HDP.

## RESULTS

A total of 7,121 SNPs associated with 119 bacterial genera were included for GM instruments. The characters of selected IVs were shown in Supplementary Table 1.

### Forward MR analyses

### HDP

**Figure 1:**
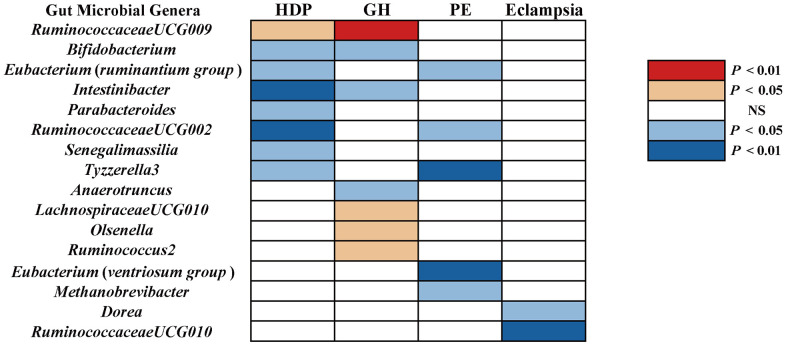
**The causal effect of gut microbial genera on RDP (GR, PE, and Eclampsia) identified at the nominal significance by using the IVW method (*P* < 0.05 / 0.01).** Red represents the risk factors for RDP, blue represents the protective factors for RDP, and white represents no causal association. RDP, hypertensive disorders in pregnancy; GR, gestational hypertension; PE, pre-eclampsia; NS, No significant association.

Results at a significant threshold of *P* < 0.05 by using the inverse-variance weighted (IVW) method were shown in Figure 1. We found a causal association of increase in *RuminococcaceaeUCG009* (*OR* = 1.18, 95%*CI*: 1.03-1.34, *P* = 0.015) and higher risk of HDP, while genetically increased in *Bifidobacterium* (*OR* = 0.81, 95%*CI*: 0.68-0.97, *P* = 0.022), *Eubacterium* (*ruminantium group*) (*OR* = 0.81, 95%*CI*: 0.69-0.96, *P* = 0.012), *Intestinibacter* (*OR* = 0.83, 95%*CI*: 0.72-0.96, *P* = 0.011), *Parabacteroides* (*OR* = 0.75, 95%*CI*: 0.57-0.99, *P* = 0.047), *RuminococcaceaeUCG002* (*OR* = 0.84, 95%*CI*: 0.74-0.96, *P* = 0.011), *Senegalimassilia* (*OR* = 0.80, 95%CI: 0.65-0.98, P = 0.033), and *Tyzzerella3* (*OR* = 0.87, 95% *CI*: 0.77-0.99, *P* = 0.039) were associated with protective effects on HDP. These causal associations, however, lost their significance when multiple comparisons were adjusted (*q* > 0.1). The *F*-statistics ranged from 142.45 to 242.46 among all the results above, excluding the weak IVs bias. Details of all the IVW results were shown in Supplementary Table 2.

### GH

**Figure 2:**
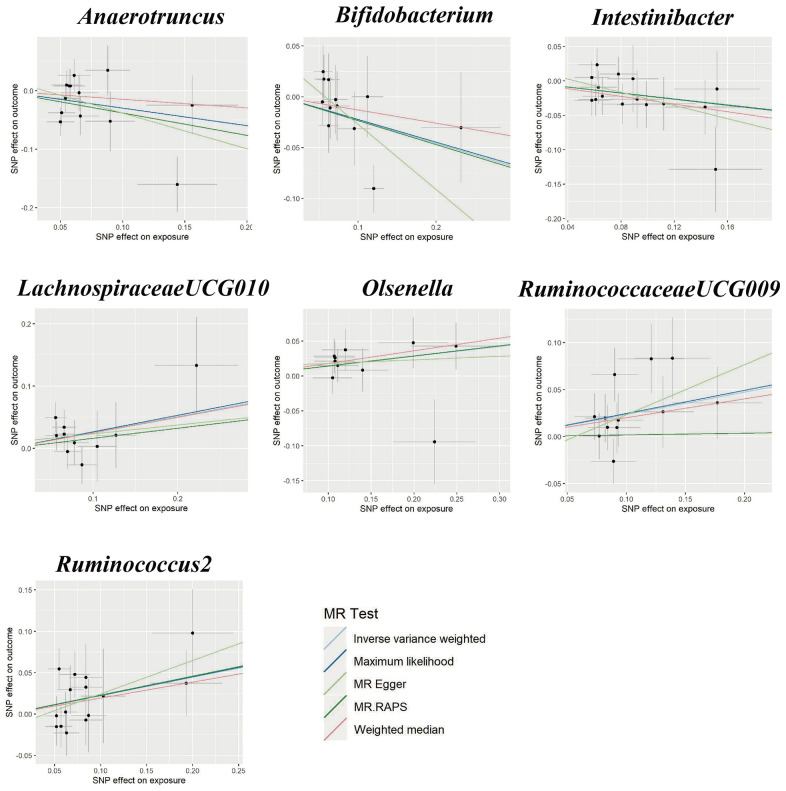
Scatter plots for the causal relationship between gut microbiota and gestational hypertension.

Using the IVW method, we found suggestive causal associations of increases in *LachnospiraceaeUCG010* (*OR* = 1.29, 95%*CI*: 1.00-1.66, *P* = 0.049), *Olsenella* (*OR* = 1.15, 95% *CI*: 1.02-1.31, *P* = 0.028), *RuminococcaceaeUCG009* (*OR* = 1.27, 95%*CI*: 1.08-1.49, *P* = 0.005), and *Ruminococcus2* (*OR* = 1.25, 95%*CI*: 1.03-1.51, *P* = 0.022) and higher risk of GH, while genetically increased in *Anaerotruncus* (*OR* = 0.74, 95%*CI*: 0.55-0.99, *P* = 0.047), *Bifidobacterium* (*OR* = 0.80, 95%*CI*: 0.65-0.98, *P* = 0.030), and *Intestinibacter* (*OR* = 0.80, 95%*CI*: 0.67-0.97, *P* = 0.023) were related to protective effects on GH (Figure 1). However, causal associations lost their significance when multiple comparisons were adjusted. Details of all the IVW results were shown in Supplementary Table 3. The *F*-statistics ranged from 144.51 to 205.87 among all the results above. Additionally, causal associations between GM and GH risk were found in more than three MR methods (Table 1 and Figure 2), including IVW, Maximum Likelihood (MaxLik), Weighted Median (WM), MR-Egger regression, and MR robust adjusted profile score (MR.RAPS).

**Table 1:** MR analyses of gut microbiota on HDP subtypes by different methods.

**Exposure**	**Outcome**	***F*-Stat**	**Inverse variance weighted**			**Maximum likelihood**			**Weighted median**			**MR.RAPS**			**MR Egger**	
			***OR* (95%*CI*)**	***P***		***OR* (95%*CI*)**	***P***		***OR* (95%*CI*)**	***P***		***OR* (95%*CI*)**	***P***		***OR* (95%*CI*)**	***P***
*Anaerotruncus*	GH	166.34	0.74(0.55,0.99)	0.047		0.74(0.58,0.95)	0.019		0.86(0.61,1.23)	0.410		0.68(0.54,0.86)	0.001		0.54(0.23,1.31)	0.203
*Bifidobacterium*	GH	139.63	0.80(0.65,0.98)	0.030		0.80(0.66,0.97)	0.026		0.88(0.65,1.19)	0.401		0.79(0.66,0.95)	0.014		0.53(0.32,0.86)	0.027
*Intestinibacter*	GH	159.39	0.80 (0.67,0.97)	0.023		0.81(0.66,0.98)	0.027		0.76(0.59,0.97)	0.028		0.80(0.66,0.98)	0.031		0.62(0.34,1.14)	0.150
*Lachnospiraceae* *UCG010*	GH	144.51	1.29(1.00,1.66)	0.049		1.31(1.01,1.69)	0.046		1.28(0.90,1.83)	0.168		1.18(0.92,1.50)	0.188		1.15(0.53,2.51)	0.731
*Olsenella*	GH	205.87	1.15(1.02,1.31)	0.028		1.16(1.02,1.32)	0.028		1.20(1.01,1.42)	0.035		1.16(1.01,1.32)	0.038		1.05(0.70,1.58)	0.810
*Ruminococcaceae* *UCG009*	GH	177.26	1.27(1.08,1.49)	0.005		1.28(1.08,1.52)	0.005		1.22(0.97,1.54)	0.085		1.20(1.03,1.41)	0.023		1.70(0.88,3.29)	0.143
*Ruminococcus2*	GH	156.71	1.25(1.03,1.51)	0.022		1.25(1.03,1.52)	0.022		1.21(0.91,1.62)	0.190		1.26(1.03,1.54)	0.026		1.50(0.95,2.37)	0.106
*Eubacterium* (*ruminantium group*)	PE	157.34	0.86(0.75,0.99)	0.045		0.86(0.74,1.00)	0.049		0.90(0.74,1.10)	0.314		0.89(0.77,1.03)	0.118		1.24(0.77,2.01)	0.391
*Eubacterium* (*ventriosum group*)	PE	160.92	0.74(0.59,0.93)	0.011		0.75(0.59,0.94)	0.014		0.72(0.53,0.98)	0.034		0.75(0.59,0.94)	0.015		0.68(0.24,1.90)	0.473
*Methanobrevibacter*	PE	179.04	0.79(0.65,0.96)	0.019		0.79(0.65,0.96)	0.020		0.80(0.63,1.02)	0.070		0.79(0.66,0.95)	0.014		0.55(0.26,1.13)	0.180
*Ruminococcaceae* *UCG002*	PE	166.26	0.80(0.65,0.98)	0.029		0.80(0.66,0.97)	0.021		0.80(0.61,1.05)	0.105		0.85(0.71,1.02)	0.075		0.90(0.52,1.55)	0.702
*Tyzzerella3*	PE	147.81	0.80(0.68,0.93)	0.003		0.79(0.68,0.93)	0.004		0.77(0.63,0.95)	0.015		0.79(0.68,0.92)	0.003		0.73(0.29,1.80)	0.506
*Dorea*	Eclampsia	174.09	0.27(0.09,0.76)	0.014		0.26(0.09,0.75)	0.014		0.46(0.10,2.14)	0.323		0.16(0.06,0.46)	0.001		1.07(0.06,20.03)	0.963
*Ruminococcaceae* *UCG010*	Eclampsia	151.49	0.20(0.07,0.58)	0.003		0.20(0.07,0.60)	0.004		0.24(0.06,1.00)	0.050		0.30(0.11,0.83)	0.020		0.20(0.01,3.67)	0.340

GH, gestational hypertension; PE, pre-eclampsia; *F*-stat, *F* statistics to detect weak instrumental variable bias; MR.RAPS, Mendelian randomization robust adjusted profile score; *OR*, odds ratio; *CI*, confidence interval; *P*, *P* value.

### PE

**Figure 3:**
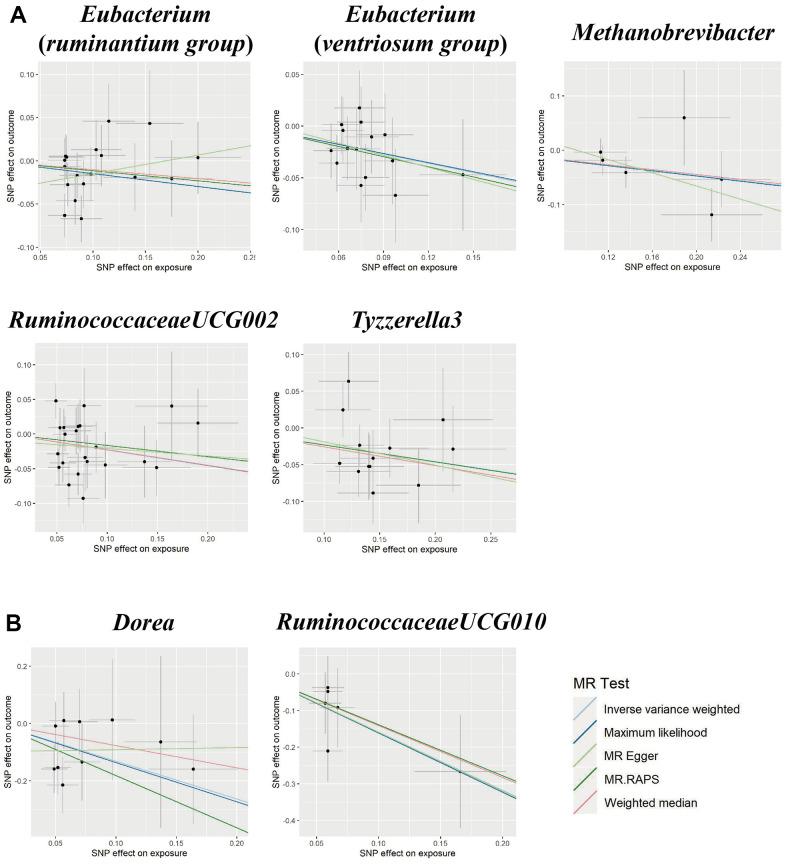
Scatter plots for the causal association between gut microbiota and (**A**) pre-eclampsia or (**B**) eclampsia.

We found five suggestive causal effects of GM on PE (*P* < 0.05, *q* > 0.1; Figure 1). Specifically, *Eubacterium*(*ruminantium group*) (*OR* = 0.86, 95%*CI*: 0.75-0.99, *P* = 0.045), *Eubacterium*(*ventriosum group*) (*OR* = 0.74, 95%*CI*: 0.59-0.93, *P* = 0.011), *Methanobrevibacter* (*OR* = 0.79, 95%*CI*: 0.65-0.96, *P* = 0.019), *RuminococcaceaeUCG002* (*OR* = 0.80, 0.65-0.98, *P* = 0.029), and *Tyzzerella3* (*OR =* 0.80, 95*%CI*: 0.68-0.93*, P* = 0.003) were negatively associated with the risk of PE. Details of all the IVW results were shown in Supplementary Table 4. The *F*-statistics ranged from 147.81 to 179.04 among all the results above. Furthermore, causal associations between GM and PE risk were found in more than three MR methods (Table 1 and Figure 3).

### Eclampsia

We found suggestive causal effects of GM on Eclampsia in two microbial genera, including *Dorea* (*OR* = 0.27, 95%*CI*: 0.09-0.76, *P* = 0.014) and *RuminococcaceaeUCG010* (*OR* = 0.20, 95%*CI*: 0.07-0.58, *P* = 0.003), which were negatively associated with the risk of eclampsia (Figure 1). Details of all the IVW results were shown in Supplementary Table 5. The *F*-statistics were 174.09 and 151.49, respectively, Furthermore, causal associations between GM and eclampsia risk were found in more than two of the MR methods (Table 1 and Figure 3).

### Sensitivity analyses

Cochran’s Q statistics showed no significant heterogeneity in selected IVs (*P* > 0.05 in IVW and MR-Egger methods, Supplementary Table 6). Both the MR-Egger intercept and the MR-PRESSO global test confirmed there is no significant directional horizontal pleiotropy (*P* > 0.05, Supplementary Table 6). Additionally, the leave-one-out analysis revealed that there are no outlier IVs that would have a significant impact on the result if retained (Supplementary Figures 1–3).

### Reverse MR analyses

We performed the reverse MR analysis to assess whether specific HDP subtypes causally affect gut microbiota to confirm the causal direction. However, all methods showed no causal relationship except for the genus *Bifidobacterium* (*P* > 0.05, Supplementary Table 7). The sensitivity analyses including Cochran’s Q test, MR-Egger regression intercept, MR-PRESSO global test, and the leave-one-out sensitivity analysis confirmed the robustness of the reverse MR results (Supplementary Table 8 and Supplementary Figures 4–6).

### Multivariable MR analyses

**Figure 4:**
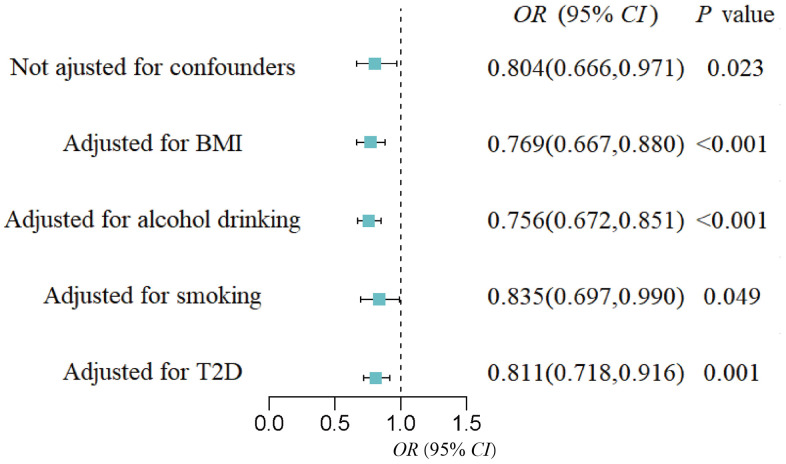
Forest plot of the causal effect of the genus *Intestinibacter* on gestational hypertension after adjusting for confounders.

MVMR analysis was performed to assess the causal effect of GM on GH, PE, and eclampsia, respectively after confounding factors were adjusted (BMI, alcohol drinking, smoking, and T2D). For the genus *Intestinibacter*, after adjusting for BMI (*OR* = 0.77, 95%*CI*: 0.67-0.88, *P* < 0.001), alcohol drinking (*OR* = 0.76, 95%*CI*: 0.67-0.85, *P* < 0.001), smoking (*OR* = 0.84, 95%*CI*: 0.70-0.99, *P* = 0.049), and T2D (*OR* = 0.81, 95%*CI*: 0.72-0.92, *P* = 0.001), *Intestinibacter* remained causally associated with GH risk (Figure 4). Detailed MVMR results of other suggestive association GM on HDP subtypes were shown in Table 2.

**Table 2:** Multivariable MR analyses of gut microbiota on HDP subtypes after adjusting confounding factors.

**Exposure**	**Outcome**	**BMI**			**Alcohol drinking**			**Smoking**			**T2D**	
		***OR* (95%*CI*)**	***P***		***OR* (95%*CI*)**	***P***		***OR* (95%*CI*)**	***P***		***OR* (95%*CI*)**	***P***
*Anaerotruncus*	GH	0.71(0.43,1.18)	0.187		0.76(0.52,1.11)	0.159		0.70(0.50,0.98)	0.035		0.91(0.71,1.15)	0.419
*Bifidobacterium*	GH	0.79(0.68,0.93)	0.004		0.76(0.63,0.91)	0.004		0.74(0.62,0.89)	0.001		0.77(0.54,1.08)	0.128
*Intestinibacter*	GH	0.77(0.67,0.88)	<0.001		0.76(0.67,0.85)	<0.001		0.84(0.70,0.99)	0.049		0.81(0.72,0.92)	0.001
*Lachnospiraceae UCG010*	GH	1.59(1.21,2.08)	0.001		1.15(0.84,1.56)	0.388		1.33(1.10,1.59)	0.003		1.13(1.06,1.19)	0.025
*Olsenella*	GH	1.14(0.93,1.42)	0.215		1.13(1.00,1.28)	0.049		1.12(1.01,1.25)	0.041		1.17(1.06,1.289)	0.003
*Ruminococcaceae UCG009*	GH	1.40(1.11,1.77)	0.005		1.28(1.04,1.57)	0.020		1.49(1.28,1.74)	<0.001		1.12(1.06,1.19)	<0.001
*Ruminococcus2*	GH	1.25(1.04,1.51)	0.021		1.18(0.94,1.47)	0.154		1.29(1.09,1.54)	0.004		1.24(0.84,1.84)	0.285
*Eubacterium* (*ruminantium group*)	PE	0.85(0.71,1.02)	0.082		0.89(0.77,1.03)	0.119		0.87(0.74,1.02)	0.085		0.81(0.65,1.02)	0.070
*Eubacterium* (*ventriosum group*)	PE	0.79(0.64,0.98)	0.026		0.73(0.63,0.85)	<0.001		0.76(0.63,0.93)	0.006		0.82(0.71,0.95)	0.007
*Methanobrevibacter*	PE	0.77(0.59,1.02)	0.067		0.77(0.66,0.91)	0.002		0.65(0.50,0.86)	0.002		0.76(0.64,0.90)	0.001
*RuminococcaceaeUCG002*	PE	1.13(0.87,1.48)	0.360		0.79(0.63,0.98)	0.034		0.80(0.65,0.98)	0.003		0.67(0.50,0.90)	0.008
*Tyzzerella3*	PE	0.76(0.69,0.83)	<0.001		0.88(0.76,1.02)	0.089		0.76(0.65,0.90)	0.001		0.87(0.21,1.20)	0.394
*Dorea*	Eclampsia	0.53(0.25,1.14)	0.106		0.30(0.12,0.72)	0.007		0.29(0.11,0.74)	0.010		0.22(0.06,0.82)	0.024
*RuminococcaceaeUCG010*	Eclampsia	0.01(0.00,0.01)	<0.001		0.31(0.09,1.03)	0.057		0.40(0.17,0.90)	0.027		20.61(1.71,247.96)	0.017

GH, gestational hypertension; PE, pre-eclampsia; *F*-stat, *F* statistics to detect weak instrumental variable bias; *OR*, odds ratio; *CI*, confidence interval; *P*, *P* value; BMI, body mass index; T2D, type 2 diabetes.

## DISCUSSION

In this multivariable MR study, we detected causal associations between eight particular bacterial genera and the risk of HDP, then replicated the analyses in specific subtypes (GH, PE, and eclampsia). Specifically, we identified suggestive causal associations of *LachnospiraceaeUCG010, Olsenella, RuminococcaceaeUCG009*, *Ruminococcus2*, *Anaerotruncus*, *Bifidobacterium*, and *Intestinibacter* with GH, of *Eubacterium* (*ruminantium group*), *Eubacterium* (*ventriosum group*), *Methanobrevibacter*, *RuminococcaceaeUCG002*, and *Tyzzerella3* with PE, as well as of *Dorea* and *RuminococcaceaeUCG010* with eclampsia. For example, our MR analyses revealed a protective effect of *Bifidobacterium* on GH. A case-control study including 170 women in early pregnancy found that the relative abundance of *Bifidobacterium* significantly decreases in HDP patients compared with the control group [17], which was consistent with the previous studies on hypertension patients in Tangshan and Henan [18, 19]. It has been reported that *Bifidobacterium* can restore intestinal barrier function by stimulating the expression of Mucins 3 [20]. The Mice infection model also supported the role of *Bifidobacterium* in maintaining barrier permeability by reducing the concentration of Shiga toxin in *enterohemorrhagic E. coli* strains [21]. Furthermore, treatment with *Bifidobacterium bifidum* significantly lowered the rates of bacterial translocation [22], and stopped the entry of GM-derived lipopolysaccharide (LPS) into blood [23], thereby reducing placental inflammation and maintaining normal blood pressure. All the evidence above supported the protective role of *Bifidobacterium* on GH.

In addition, we also found the genus *Intestinibacter* to be associated with a lower risk of GH. There have been relatively few previous studies on *Intestinibacter*, but observational study and animal model have both reported the role of *Intestinibacter* in producing butyrate [24, 25], which is a short chain fatty acid (SCFA) metabolized by GM. Placental inflammation and angiogenic factors played the central role in affecting blood pressure in pregnancy, and macrophages are the key regulator [26]. *In vivo* and *in vitro* experiments found that butyrate significantly reduces the effects of LPS to promote macrophage 1 polarization and inhibit macrophage 2 polarization, thereby reducing blood pressure [27, 28]. Furthermore, Jin et al. reported that butyrate promotes the effect on macrophage autophagy by decreasing autophagy receptors like P62 level and elevating LC3-II/LC3-I ratio, thus alleviating PE symptoms in rats [29]. The higher abundance of butyrate has been reported to decrease the risk of insulin resistance and type 2 diabetes(T2D) [30] and T2D is positively associated with blood pressure, suggesting that the effect of *Intestinibacter* on GH may be biased by T2D. But our multivariable MR analysis demonstrated that after adjusting T2D, the protective effect remained, which excluding the influence of confounding factors. Meanwhile, *Bifidobacterium* [9], *Eubacterium* (*ruminantium group*) [31], *Tyzzerella3* [32], and *Dorea* [33] have also been reported to produce SCFA with the function to effectively reduce blood pressure [34, 35], which consistent with our MR results that the increased abundance of those gut microbiota were related to the lower risk of HDP.

Interestingly, the HDP-associated gut microbial genera identified by our MR analyses varied in different subtypes. For example, we didn’t find the relationships between *Bifidobacterium* and other HDP subtypes except for GH, which was consistent with another MR analysis on the gut microbiota and adverse pregnancy outcomes [16], while Li et al. reported the opposite result [36]. It may, because of the different pathogenesis, biochemical index, and degree of organ damage in GH, PE, and eclampsia. Additionally, our study demonstrated that *RuminococcaceaeUCG009* increases the risk of GH, while *RuminococcaceaeUCG002* and *RuminococcaceaeUCG010* were found to be protective factors to PE and eclampsia, respectively. Some genera of *Ruminococcaceae* are beneficial SCFA-producing bacteria that could not only power the intestinal epithelial cells [37] but also reduce proinflammatory cytokine by monocytes [38]. For example, in population-based studies, the abundance of genus *RuminococcaceaeUCG002* was found to have beneficial implications for host glucose homeostasis and lipid metabolism [39, 40] as well as *RuminococcaceaeUCG010* was found to be fewer in hypertension patients compared with health group [41]. However, *RuminococcaceaeUCG009* was found to be positively correlated with the production of inflammatory factors and LPS in serum thus may cooperatively contribute to HDP, which supported our result [42]. Thus, our findings opened up new possibilities for understanding the differences in gut microbial genera mediating mechanisms in various subtypes of HDP. Considering the different effects of the same gut microbial genera (e.g., *Ruminococcaceae*) on human blood pressure, further RCTs at a more specific species level are needed to support this finding.

The present study has some strengths. It was the first multivariable MR analysis to explore the causal relationship between GM and HDP subtypes and find the difference of causal-related GM in GH, PE, and eclampsia. The findings would facilitate the targeted prevention and treatment of different subtypes. Secondly, our study was based on the largest GWAS summary datasets to date, along with bidirectional MR, multivariable MR analyses, and several sensitivity analyses, which indicates the robustness of our findings. Thirdly, confounding variables and reverse causation were less likely to have an impact on the causal inference by using the MR design.

Our analysis still has several limitations. Firstly, the significance threshold of exposure IVs was set at 1e-05 because of insufficient IVs under genome-wide significance. However, IVs with *F*-statistics < 10 were excluded to avoid the weak instrumental bias. Secondly, MR analyses could only conduct at the bacterial genus level rather than at a more specific species level because of limited 16S rRNA sequencing resolution. Thirdly, our research was unable to provide further mechanisms for the distinct gut microbiota taxa associated with GH, PE, and eclampsia which need subsequent functional studies to elucidate.

In conclusion, by performing bidirectional multivariable MR analyses on GWAS summary data, this study explored the causal relationship between GM and different subtypes of HDP. Our findings may offer a new strategy for prevention and treatment in different HDP subtypes by targeting the gut microbiota and provide novel insights to understand the mechanism of HDP.

## MATERIALS AND METHODS

### Data sources

GWAS summary statistics for GM were obtained from the Microbiome Genome (MiBioGen) Consortium which consisted of 24 multiple ancestry cohorts including 18,340 participants [43]. After extracting DNA from fecal samples, data were generated by 16S rRNA gene sequencing in the Illumina platform. Setting SILVA as the reference, all the data were annotated to genus and higher levels to profile the microbial composition [44].

According to the pathogenesis, biochemical index, and degree of organ damage, HDP could be divided into five subtypes including gestational hypertension (GH), pre-eclampsia (PE), eclampsia and so on [45]. In this study, firstly we tested the whole HDP group and then primarily focused on GH, PE, and eclampsia patients because they are major or most serious HDP subtypes. GWAS summary statistics for HDP were extracted from the FinnGen Consortium and updated in 2023 [46]. Briefly, the study for HDP included 123,579 female subjects (7,686 cases and 115,893 controls) covering a total of 16,379,784 SNPs. The genetic association datasets consisted of 118,990 pregnant women (4,255 cases and 114,735 controls) with GH, 118,291 pregnant women (3,556 cases and 114,735 controls) with PE, and 115,025 pregnant women (290 cases and 114,735 controls) with eclampsia, respectively. Association analysis was conducted with sex, age, genotyping batch, and 10 principal components corrected as covariates. Detailed information on exposure and outcome GWAS datasets were summarized in Supplementary Table 9.

### Instrumental variables

**Figure 5:**
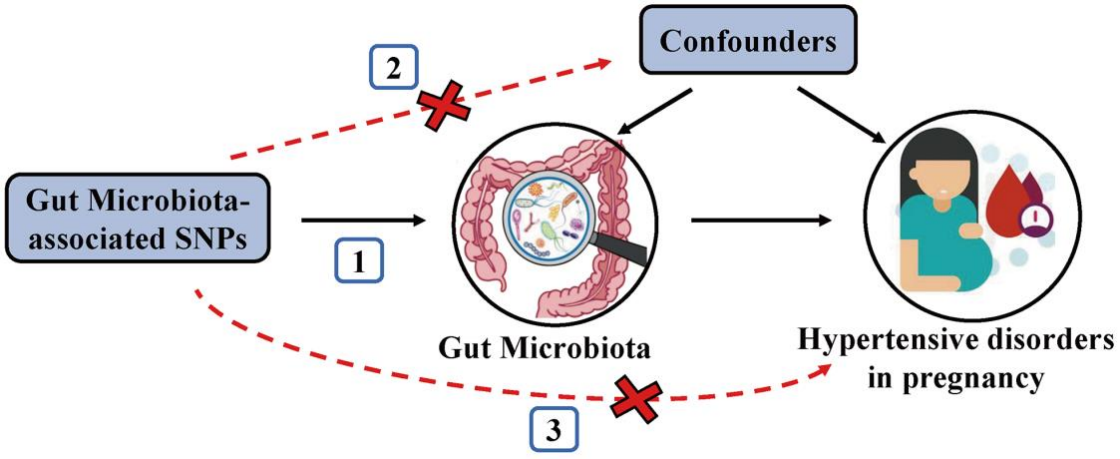
**Schematic representation of the MR analysis.** The three assumptions of MR are as follows: (1) Instrumental variables must be associated with gut microbiota, (2) Instrumental variables must not be associated with confounders; and (3) Instrumental variables must influence hypertensive disorders in pregnancy only through gut microbiota, not through other pathways.

To satisfy the three key assumptions of MR analysis (Figure 5), five steps were applied to select the optimal IVs: 1) SNPs under a locus-wide significance threshold of *P* < 1e-05 were obtained as potential IVs related to exposure [14]. 2) PLINK clumping method (*r*^2^ < 0.001, clump window < 10,000 kb) was performed to ensure the IVs were independent [47]. 3) SNPs with minor allele frequency < 0.01 and palindromic SNPs were excluded. 4) The proxy SNPs (*r*^2^ > 0.8) were selected based on European population data in the 1000 Genome project after removing the SNPs closely related to the outcome phenotype (*P* < 5e-08) [48]. 5) SNPs with *F*-statistics < 10 were eliminated to avoid weak IV bias [49].

### Statistical analyses

We used the inverse-variance weighted (IVW) method as the primary MR analysis to detect the causal associations between exposure (GM) and outcomes (HDP, GH, PE, and eclampsia). The IVW method calculates the total causal effect by using the weighted linear regression model combined with the weight coefficient, under the condition that the intercept is zero [50]. IVW results were corrected for multiple comparisons applying the *q*-value procedure (*q* < 0.1), while *P* < 0.05 but *q* > 0.1 was considered to have a suggestive association [51].

Several MR methods including Maximum Likelihood (MaxLik), Weighted Median (WM), MR-Egger regression, and MR robust adjusted profile score (MR.RAPS) were also conducted to test the robustness of our study. MaxLik estimates the parameter values that have the greatest likelihood of leading to a particular outcome by using the known sample. Its standard error would be lower than IVW when heterogeneity and horizontal pleiotropy do not exist [52]. WM improves the power of causal effect detection based on the assumption that up to 50% of IVs are valid [53]. MR-Egger regression method could identify and correct pleiotropy, but the estimation accuracy will be very low unless using a larger sample size [54]. MR.RAPS applies robust estimates to correct for systematic and idiosyncratic pleiotropy, the results of which are unbiased even though weak IVs exist [55].

Cochran’s IVW Q statistics and leave-one-out analysis were used to identify potential heterogeneous IVs. MR-Egger intercept and MR Pleiotropy RESidual Sum and Outlier (MR-PRESSO) global test were conducted to test whether directional horizontal pleiotropy is driving the results of MR analyses [56, 57].

Reverse MR analysis was used to confirm the causal direction. The methods were similar to those of forward MR except for setting exposures as HDP subtypes and outcome as GM. Finally, we conducted multivariable MR (MVMR) analyses considering the possible confounders which may affect the outcome. The confounders including BMI (IEU number: ukb-b-19953), alcohol drinking (IEU number: ukb-b-5779), smoking (IEU number: ieu-b-4877), and T2D (IEU number: ebi-a-GCST006867).

**Figure 6:**
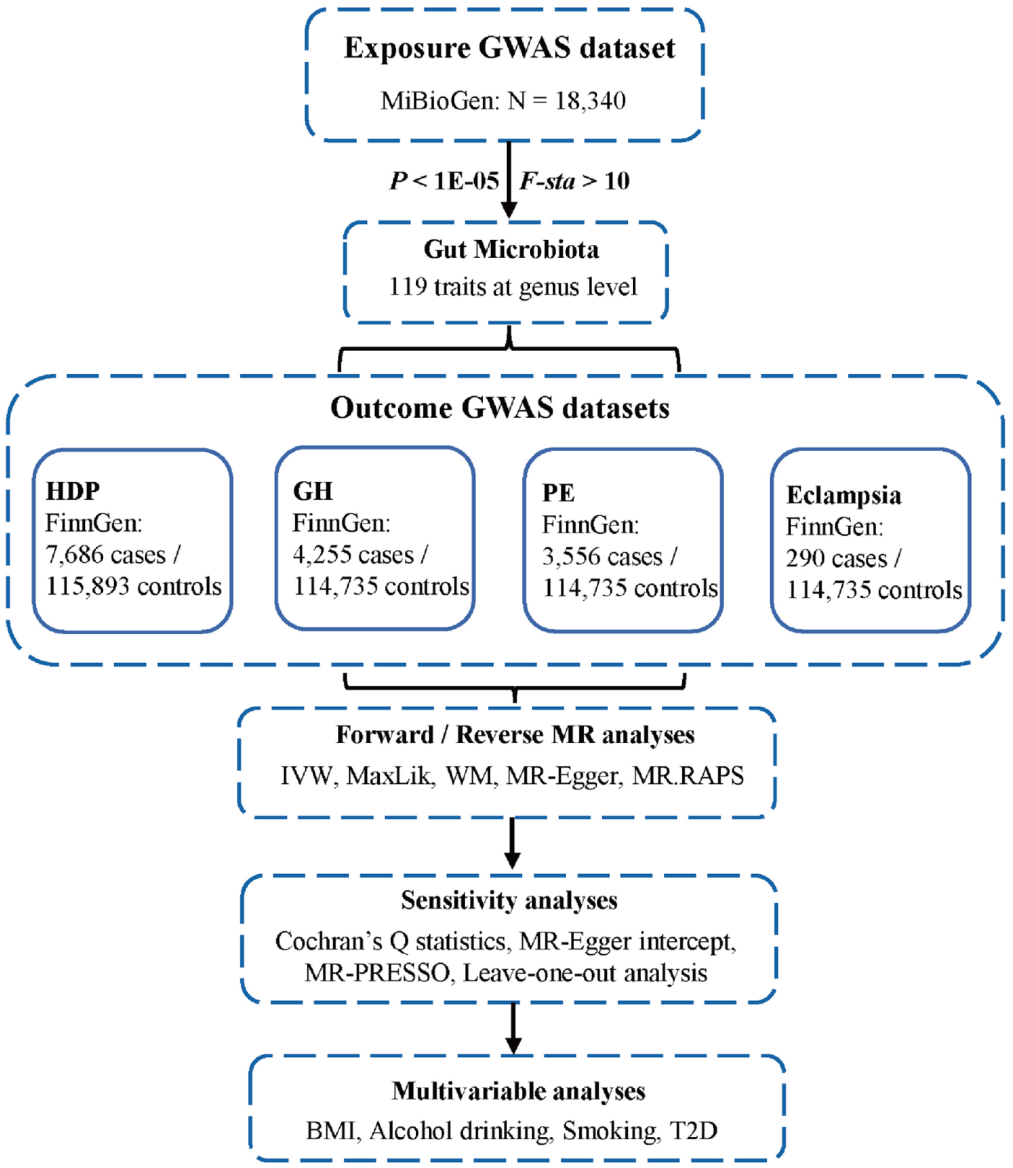
**Flowchart of this study.** GWAS, genome-wide association studies; HDP, hypertensive disorders in pregnancy; GH, gestational hypertension; PE, pre-eclampsia; MR, Mendelian randomization; IVW, inverse-variance weighted; MaxLik, maximum likelihood; WM, weighted median; MR.RAPS, MR robust adjusted profile score; MR-PRESSO, MR Pleiotropy RESidual Sum and Outlier; BMI, body mass index; T2D, type 2 diabetes.

The flowchart of this study was shown in Figure 6. All MR analyses were performed by the packages “TwoSampleMR”, “MRPRESSO”, and “qvalue” in R software.

### Consent for publication

All the authors endorsed the publication of the manuscript.

## Supplementary Material

Supplementary Figures

Supplementary Table 1

Supplementary Table 2

Supplementary Table 3

Supplementary Table 4

Supplementary Table 5

Supplementary Tables 6-9
