# Supplementary material for: Gut microbiota and hypertensive disorders in pregnancy: evidence from the Mendelian randomization study
Source: Aging (Albany NY). 2023 Sep 11;15(17):9105–27. doi: 10.18632/aging.205019 (PMC10522390; doi:10.18632/aging.205019)
Supplement: Supplementary Figures [file aging-15-205019-s001.pdf]

SUPPLEMENTARY FIGURES

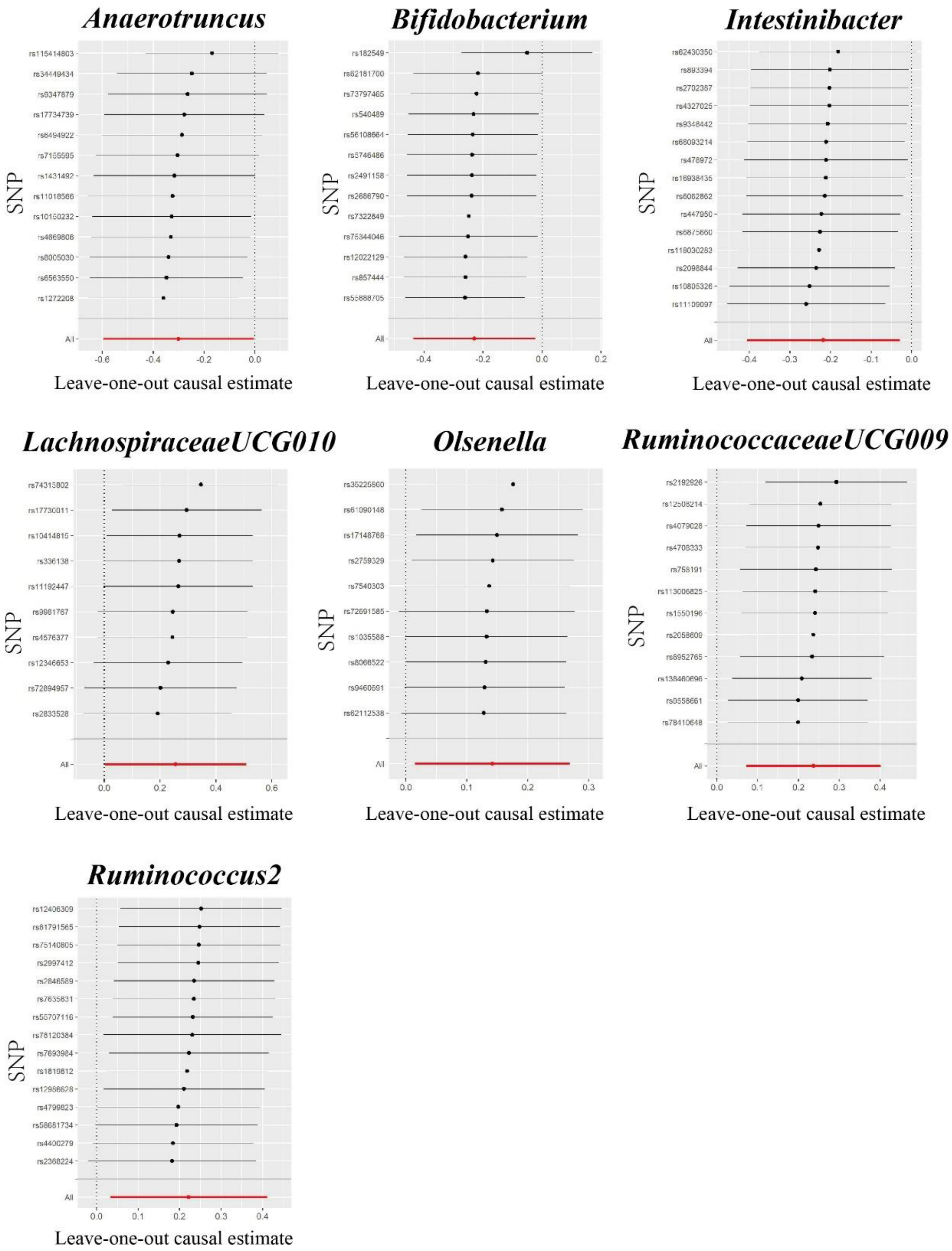

Supplementary Figure 1. Leave-one-out plots for the causal association between gut microbiota and GH in forward MR analyses.

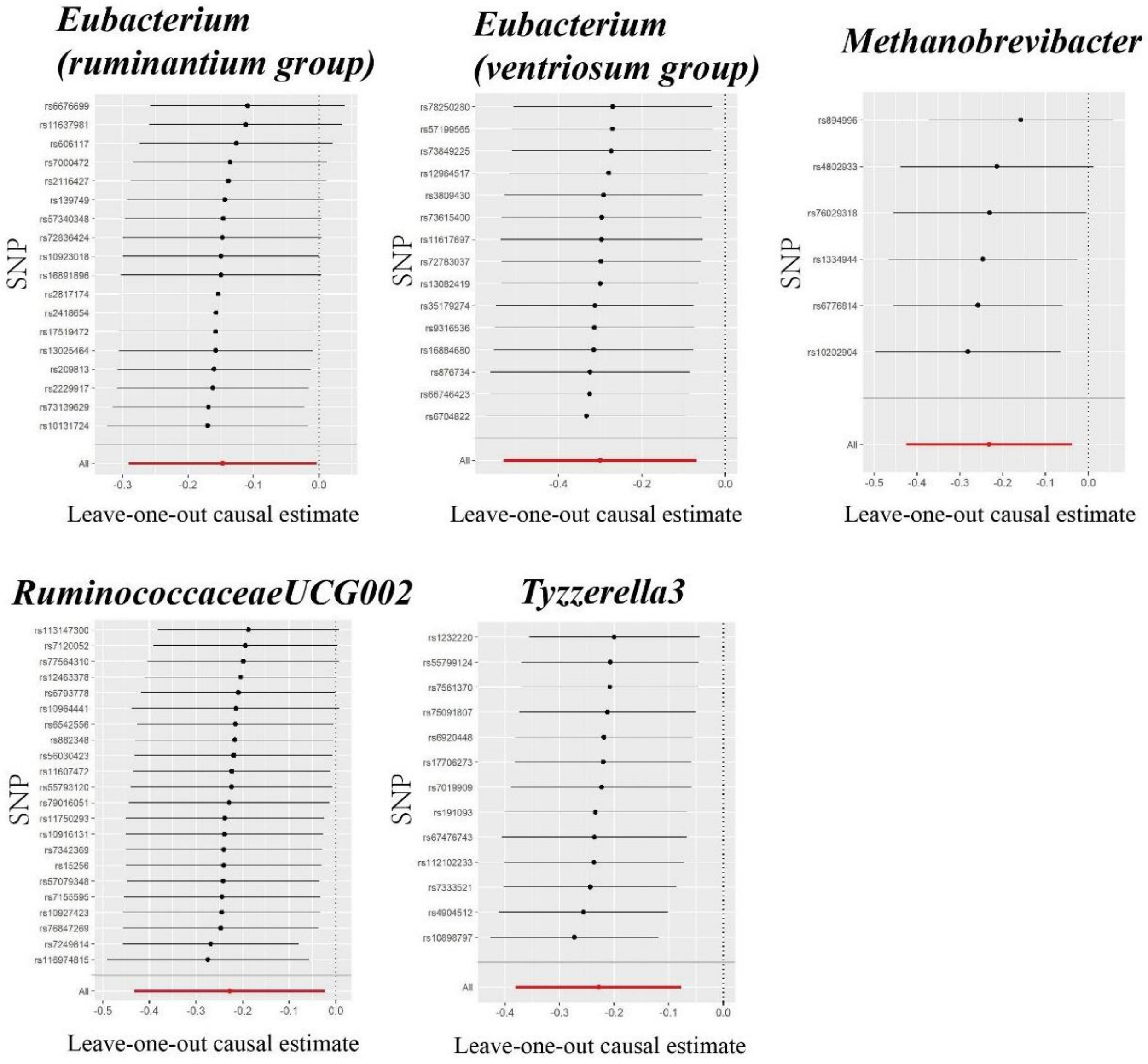

Supplementary Figure 2. Leave-one-out plots for the causal association between gut microbiota and PE in forward MR analyses.

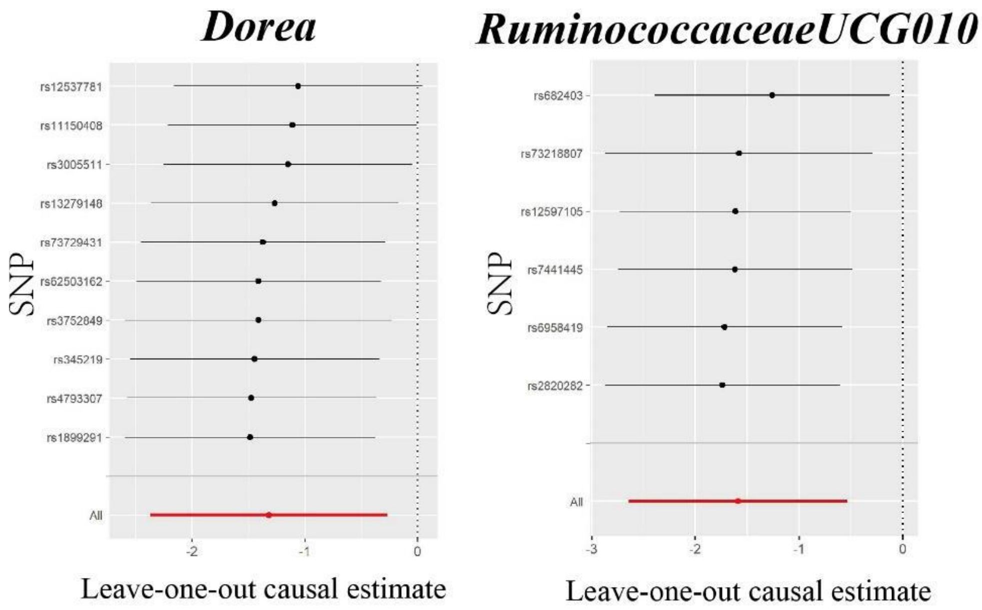

Supplementary Figure 3. Leave-one-out plots for the causal association between gut microbiota and eclampsia in forward MR analyses.

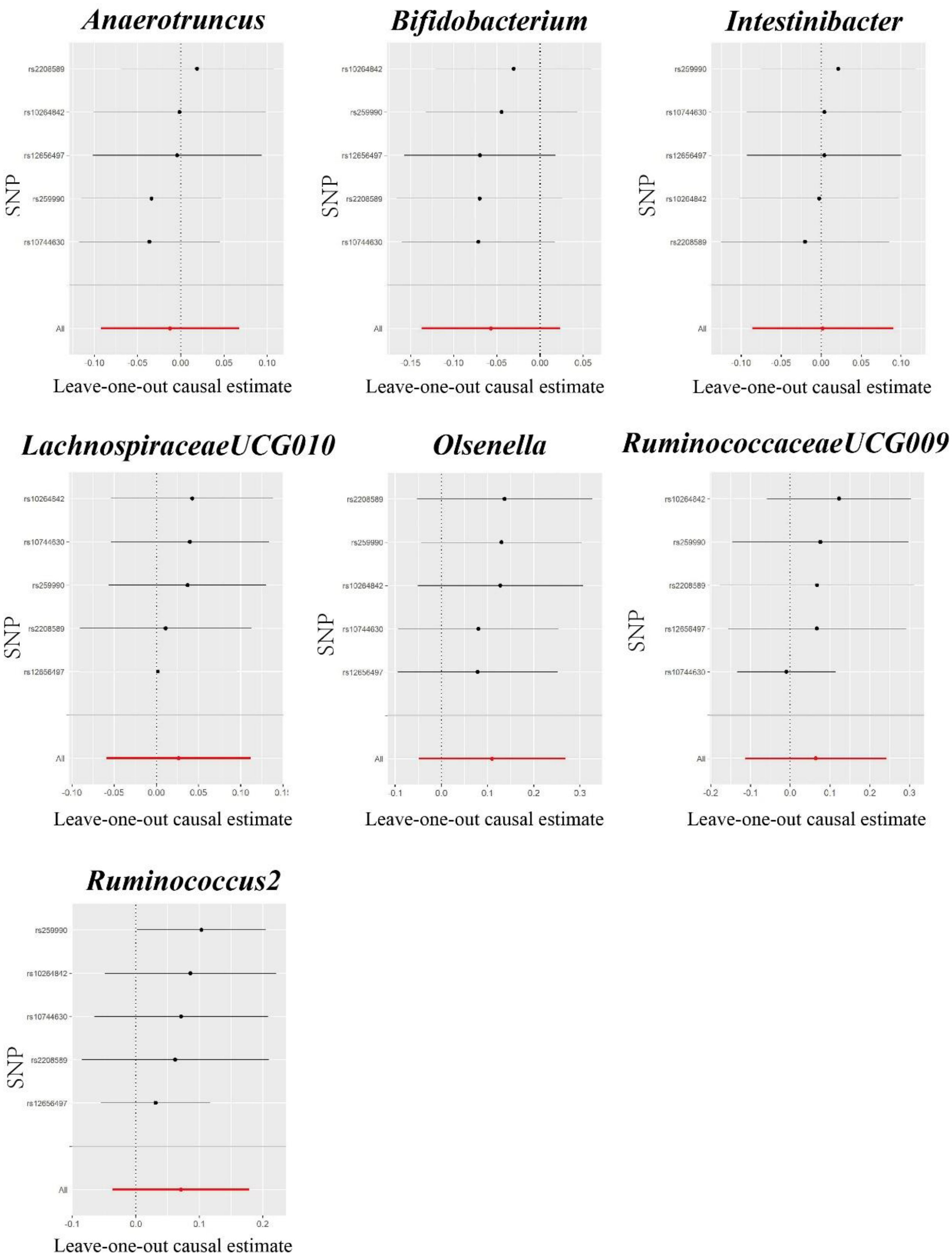

Supplementary Figure 4. Leave-one-out plots for the causal association between gut microbiota and GH in reverse MR analyses.

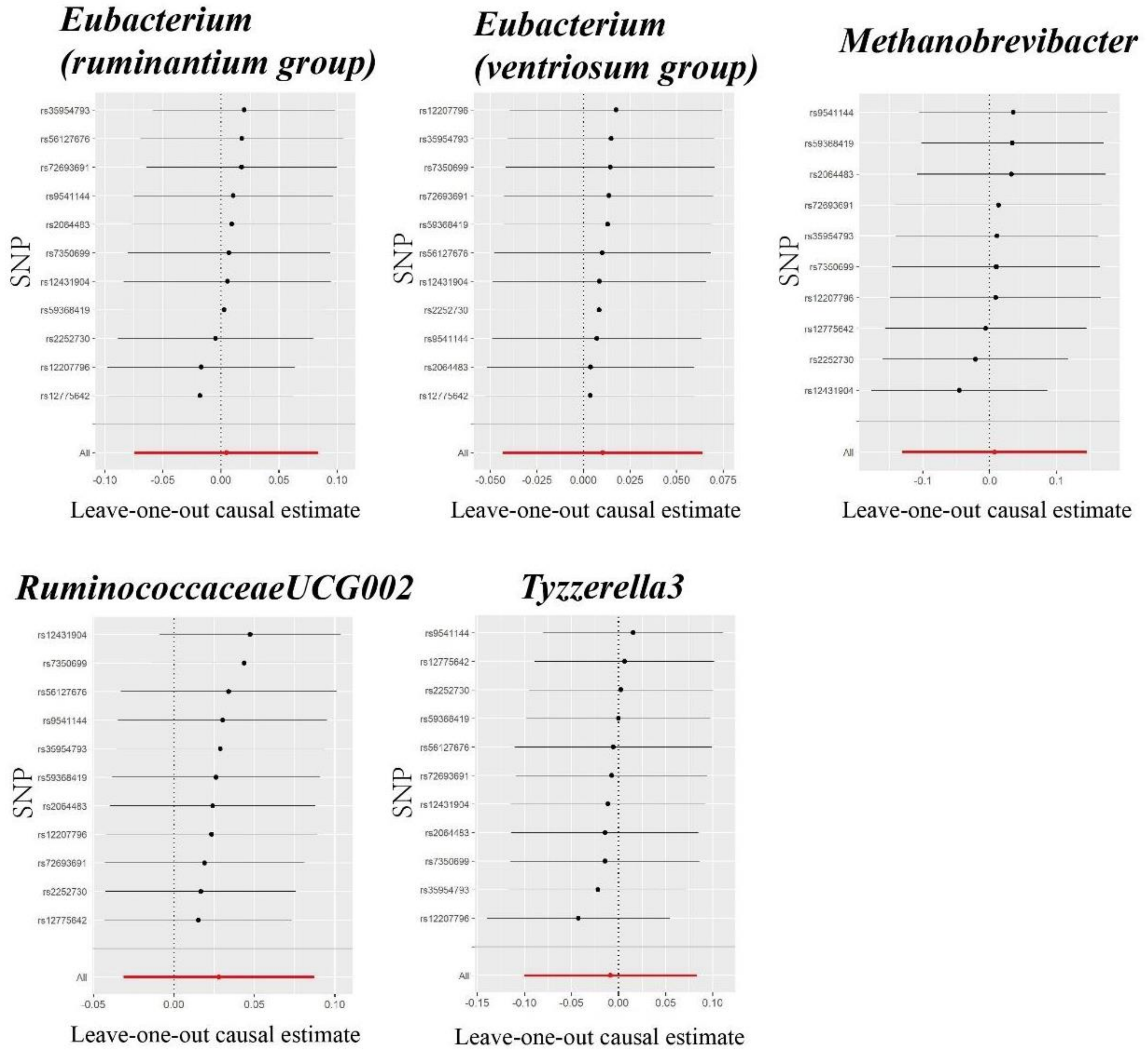

Supplementary Figure 5. Leave-one-out plots for the causal association between gut microbiota and PE in reverse MR analyses.

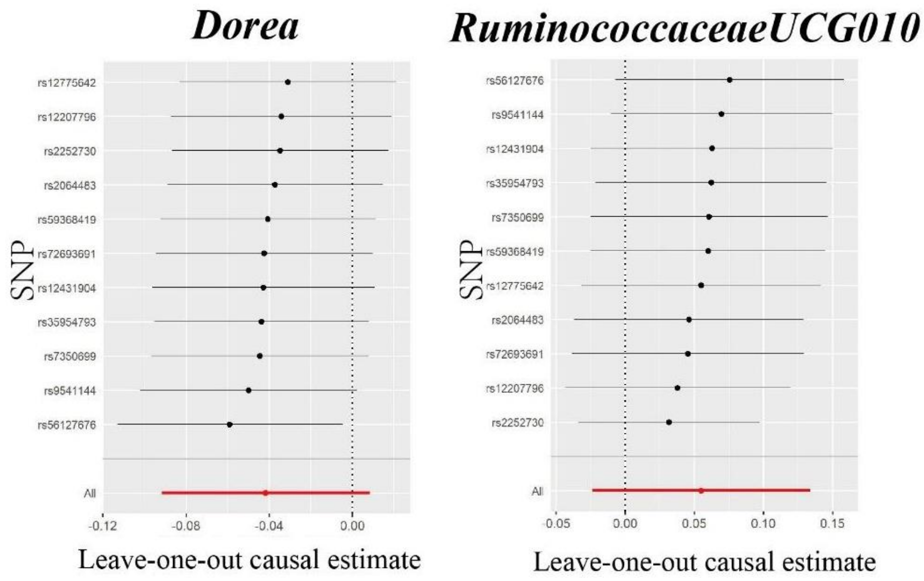

Supplementary Figure 6. Leave-one-out plots for the causal association between gut microbiota and eclampsia in reverse MR analyses.
