# Supplementary material for: Gut microbiota and hypertensive disorders in pregnancy: evidence from the Mendelian randomization study
Source: Aging (Albany NY). 2023 Sep 11;15(17):9105–27. doi: 10.18632/aging.205019 (PMC10522390; doi:10.18632/aging.205019)
Supplement: Supplementary Tables 6-9 [file aging-15-205019-s007.pdf]

Supplementary Table 6. Tests for detecting horizontal and directional pleiotropy in forward MR analysis.

Exposure		MR-PRESSO global test		MR-Egger intercept pEgger			Cochran's Q test	
		MR-PRESSO RSSobs	*P* value	Egger-intercept	Standard Error	*P* value	IVW (*P*)	MR-Egger (*P*)
GH	*RuminococcaceaeUCG009*	12.846	0.490	-0.298	0.033	0.384	0.457	0.440
GH	*Ruminococcus2*	13.843	0.624	-0.016	0.018	0.405	0.589	0.571
GH	*Intestinibacter*	9.643	0.880	0.022	0.025	0.396	0.865	0.864
GH	*Olsenella*	7.423	0.773	0.013	0.028	0.660	0.696	0.622
GH	*Bifidobacterium*	20.512	0.201	0.037	0.021	0.102	0.307	0.467
GH	*Anaerotruncus*	22.391	0.108	0.022	0.031	0.481	0.011	0.093
GH	*LachnospiraceaeUCG010*	9.269	0.572	0.009	0.030	0.769	0.613	0.521
PE	*Tyzzerella3*	14.042	0.493	0.013	0.065	0.847	0.422	0.344
PE	*Eubacteriumventriosumgroup*	6.553	0.978	0.007	0.039	0.865	0.972	0.956
PE	*Methanobrevibacter*	6.128	0.555	0.055	0.053	0.357	0.493	0.505
PE	*RuminococcaceaeUCG002*	28.348	0.221	-0.009	0.021	0.650	0.206	0.174
PE	*Eubacteriumruminantiumgroup*	18.405	0.507	-0.036	0.023	0.140	0.484	0.578
Eclampsia	*RuminococcaceaeUCG010*	3.664	0.801	0.001	0.105	0.991	0.746	0.609
Eclampsia	*Dorea*	8.411	0.679	-0.098	0.098	0.348	0.649	0.660

Supplementary Table 7. Reverse MR analyses of gut microbiota on HDP subtypes by different methods.

Exposure	Outcome	Inverse variance weighted		Maximum likelihood		Weighted median		MR.RAPS		MR Egger	
		β (95%*CI*)	*P*	β (95%*CI*)	*P*	β (95%*CI*)	*P*	β (95%*CI*)	*P*	β (95%*CI*)	*P*
GH	*Anaerotruncus*	0.006 (-0.041,0.053)	0.795	0.006 (-0.037,0.050)	0.773	-0.038 (-0.097,0.021)	0.207	0.011 (-0.031,0.054)	0.61	-0.099 (-0.283,0.085)	0.31
GH	*Bifidobacterium*	-0.083 (-0.160,-0.007)	0.033	-0.926 (-0.141,-0.044)	<0.001	-0.027 (-0.089,0.036)	0.405	-0.085 (-0.130,-0.041)	<0.001	0.140 (-0.151,0.432)	0.359
GH	*Intestinibacter*	0.003 (-0.046,0.053)	0.893	0.004 (-0.047,0.054)	0.891	-0.012 (-0.077,0.052)	0.731	-0.002 (-0.051,0.048)	0.939	-0.031 (-0.226,0.163)	0.755
GH	*Lachnospiraceae*	0.002 (-0.047,0.051)	0.943	0.002 (-0.047,0.051)	0.941	-0.015 (-0.084,0.055)	0.674	-0.003 (-0.051,0.045)	0.896	-0.039 (-0.236,0.158)	0.706
GH	*Olsenella*	-0.013 (-0.012,0.090)	0.798	-0.014 (-0.106,0.078)	0.762	0.043 (-0.086,0.171)	0.514	-0.006 (-0.094,0.083)	0.901	0.221 (-0.182,0.624)	0.296
GH	*Ruminococcaceae*	0.004 (-0.060,0.068)	0.901	0.004 (-0.060,0.069)	0.899	-0.002 (-0.090,0.087)	0.973	0.005 (-0.059,0.069)	0.871	-0.063 (-0.322,0.195)	0.637
GH	*Ruminococcus2*	0.017 (-0.038,0.072)	0.546	0.018 (-0.028,0.064)	0.435	-0.026 (-0.091,0.039)	0.43	0.012 (-0.032,0.055)	0.603	-0.046 (-0.267,0.176)	0.692
PE	*Eubacterium (ruminantium group)*	0.005 (-0.074,0.084)	0.909	0.005 (-0.073,0.082)	0.903	-0.018 (-0.124,0.088)	0.783	0.003 (-0.074,0.081)	0.81	-0.047 (-0.305,0.211)	0.73
PE	*Eubacterium (ventriosum group)*	0.010 (-0.043,0.064)	0.703	0.011 (-0.044,0.065)	0.703	0.013 (-0.054,0.081)	0.703	0.001 (-0.054,0.057)	0.969	-0.040 (-0.208,0.129)	0.657
PE	*Methanobrevibacter*	0.007 (-0.131,0.146)	0.918	0.008 (-0.118,0.133)	0.902	-0.010 (-0.185,0.165)	0.909	0.003 (-0.120,0.125)	0.967	0.218 (-0.324,0.761)	0.453
PE	*Ruminococcaceae UCG002*	0.028 (-0.031,0.087)	0.354	0.029 (-0.024,0.081)	0.283	0.022 (-0.048,0.093)	0.54	0.003 (-0.048,0.055)	0.195	-0.082 (-0.262,0.097)	0.392
PE	*Tyzzerella3*	-0.008 (-0.100,0.083)	0.856	-0.009 (-0.102,0.085)	0.851	-0.019 (-0.143,0.105)	0.764	-0.019 (-0.112,0.074)	0.692	0.121 (-0.171,0.412)	0.439
Eclampsia	*Dorea*	-0.002 (-0.021,-0.017)	0.821	-0.002 (-0.022,0.017)	0.817	0.002 (-0.024,0.029)	0.859	-0.002 (-0.023,0.018)	0.825	0.006 (-0.053,0.065)	0.847
Eclampsia	*Ruminococcaceae UCG010*	-0.003 (-0.025,0.019)	0.78	-0.003 (-0.027,0.019)	0.78	0.003 (-0.023,0.030)	0.808	-0.003 (-0.027,0.021)	0.795	0.010 (-0.055,0.076)	0.767

Supplementary Table 8. Tests for detecting horizontal and directional pleiotropy in reverse MR analysis.

Exposure	Outcome	MR-PRESSO global test		MR-Egger intercept pEgger			Cochran's Q test	
		MR-PRESSO RSSobs	*P* value	Egger-intercept	Standard Error	*P* value	IVW (*P*)	MR-Egger (*P*)
GH	*RuminococcaceaeUCG009*	14.827	0.075	0.059	0.081	0.521	0.041	0.038
GH	*Ruminococcus2*	10.731	0.183	0.057	0.042	0.265	0.114	0.204
GH	*Intestinibacter*	1.994	0.881	-0.014	0.038	0.733	0.874	0.781
GH	*Olsenella*	2.715	0.786	0.082	0.068	0.314	0.771	0.945
GH	*Bifidobacterium*	4.230	0.618	0.027	0.034	0.483	0.601	0.550
GH	*Anaerotruncus*	7.333	0.364	0.019	0.037	0.636	0.329	0.238
GH	*LachnospiraceaeUCG010*	3.951	0.673	0.023	0.036	0.575	0.623	0.526
PE	*Tyzzerella3*	12.231	0.449	-0.022	0.024	0.384	0.432	0.415
PE	*Eubacteriumventriosumgroup*	3.393	0.987	0.008	0.014	0.555	0.985	0.981
PE	*Methanobrevibacter*	14.515	0.255	-0.033	0.042	0.453	0.237	0.215
PE	*RuminococcaceaeUCG002*	16.654	0.203	0.019	0.015	0.236	0.192	0.240
PE	*Eubacteriumruminantiumgroup*	13.034	0.397	0.009	0.021	0.690	0.370	0.301
Eclampsia	*RuminococcaceaeUCG010*	22.031	0.064	0.019	0.020	0.368	0.053	0.057
Eclampsia	*Dorea*	8.979	0.688	-0.018	0.013	0.185	0.709	0.824

Supplementary Table 9. Detailed information for genome-wide association studies involved in the present mendelian randomization study.

Variable	Consortium or study	Sample size	Journal	Year	Cohort	Nation of cohort	Number of samples
**Gut microbiota**	MiBioGen	18,340	Nat Genet.	2021	BSPSPC	Germany	721
					CARDIAw	USA	257
					COPSAC	Denmark	380
					DanFunD16	Denmark	2,396
					FGFP	Belgian	2,259
					FOCUS	Germany	960
					GEM_HCE_v12	Canada	378
					GEM_HCE_v24	Canada	203
					GEM_ICHIP_HCE	Canada	662
					GenR	The Netherlands	1,328
					HCHS/SOL	USA	1,097
					KSCS	South Korea	811
					LLD	The Netherlands	875
					METSIM	Finland	522
					MIBS	The Netherlands	80
					NGRC	USA	77
					NTR	The Netherlands	279
					PNP	Israel	481
					POPCOL	Sweden	134
					RS3	The Netherlands	1,220
					SHIP	Germany	996
					SHIP-TREND	Germany	905
					TwinsUK	UK	1,205
**HDP**	FinnGen	1,22,421		2021	FinnGen	European	7,686 cases/114,735 controls
**GH**	FinnGen	1,18,990		2021	FinnGen	European	4,255 cases/114,735 controls
**PE**	FinnGen	1,18,291		2021	FinnGen	European	3,556 cases/114,735 controls
**Eclampsia**	FinnGen	1,15,025		2021	FinnGen	European	290 cases/114,735 controls
